# Association of maternal triglyceride responses to thyroid function in early pregnancy with gestational diabetes mellitus

**DOI:** 10.3389/fendo.2022.1032705

**Published:** 2022-11-28

**Authors:** Chen Zhang, Lilian Bai, Kuan Sun, Guolian Ding, Xinmei Liu, Yanting Wu, Hefeng Huang

**Affiliations:** ^1^ International Peace Maternity and Child Health Hospital, School of Medicine, Shanghai Jiao Tong University, Shanghai, China; ^2^ Institute of Reproduction and Development, Obstetrics and Gynecology Hospital of Fudan University, Shanghai, China; ^3^ Department of Fetal Medicine and Prenatal Diagnosis Center, Shanghai First Maternity and Infant Hospital, Tongji University School of Medicine, Shanghai, China; ^4^ Research Units of Embryo Original Diseases, Chinese Academy of Medical Sciences (No. 2019RU056), Shanghai, China

**Keywords:** TG, FT4, gestational diabetes mellitus, pregnancy, risk factors

## Abstract

**Introduction:**

The prevalence of Gestational Diabetes Mellitus (GDM) is increasing globally, and high levels of triglyceride (TG) and low levels of free thyroxine (FT4) in early pregnancy are associated with an increased risk of GDM; however, the interaction and mediation effects remain unknown. The aim of the present study is to examine the impact of FT4 and TG combined effects on the prevalence of GDM and the corresponding casual paths among women in early pregnancy.

**Materials and methods:**

This study comprised 40,156 pregnant women for whom early pregnancy thyroid hormones, fasting blood glucose as well as triglyceride were available. GDM was diagnosed using a 2-hour 75-g oral glucose tolerance test (OGTT) according to the American Diabetes Association guidelines, and the pregnant women were grouped and compared according to the results.

**Results:**

An L-shaped association between FT4 and GDM was observed. The prevalence of GDM increased with increasing TG levels. After accounting for multiple covariables, the highest risk for GDM was found among pregnant women of lower FT4 with the highest TG concentrations (odds ratio, 2.44, 95% CI, 2.14 to 2.80; *P*<0.001) compared with mothers of higher FT4 with the TG levels in the lowest quartile (Q1). There was a significant interaction effect of maternal FT4 and TG levels on the risk for GDM (*P* for interaction = 0.036). The estimated proportion of the mediating effect of maternal TG levels was 21.3% (95% CI, 15.6% to 36.0%; *P* < 0.001). In the sensitivity analysis, the mediating effect of TG levels was stable across subgroups.

**Conclusion:**

This study demonstrated an L-shaped association between maternal FT4 levels and GDM and the benefit of low TG levels, in which maternal TG levels act as an important mediator in this association. Our findings suggested that pregnant women who treat hypothyroidism should also reduce triglycerides levels in early pregnancy to prevent GDM development.

## Introduction

Gestational diabetes mellitus (GDM) is defined as glucose intolerance that is first detected during pregnancy and is a very common complication of pregnancy, occurring in 3% to 9% of pregnant women (1). The occurrence of GDM has adverse effects on pregnant women and their newborns. For example, mothers with GDM are more likely to suffer from glucose metabolism disorders during long-term follow-up after pregnancy, and their children are more likely to be obese when they grow up (2, 3). Gestational diabetes is usually diagnosed between 24 and 28 weeks of gestation, but if people at high risk of GDM are identified early, the risk can be reduced through lifestyle and/or medical interventions (4). However, one intervention may not be appropriate for all high-risk women, although a healthy lifestyle can certainly be recommended for all pregnant women before and during pregnancy (5, 6). Therefore, many researchers are committed to exploring increasingly effective routine biochemical markers in early pregnancy, which is of great significance for the early identification and targeted guidance of groups at high risk for GDM.

Normal thyroid function during pregnancy is critical for both the mother and fetus. However, thyroid dysfunction is a common endocrine disorder in women, affecting approximately 4% of pregnant women (7). Thyroid hormones can promote islet cell function and proliferation and play important roles in regulating glucose homeostasis (8, 9). Studies have shown that patients with both clinical and subclinical hypothyroidism are insulin resistant (10). Hypothyroidism not only affects the growth and development of the fetus but also may cause a variety of pregnancy complications. Subclinical hypothyroidism and isolated hypothyroxinaemia are both associated with adverse obstetric outcomes (11). Lower thyroid hormone levels in early pregnancy are associated with an increased risk of GDM (12). In addition, thyroid hormones also play an important role in lipid synthesis, mobilization and metabolism (13, 14). Hypothyroid patients can develop hypertriglyceridemia and hypercholesterolemia, which may be due in part to the increased hepatic output of very low-density lipoprotein (13). Additionally, patients with thyroid stimulating hormone (Thyroid-stimulating hormone, TSH) levels in the upper limit of the normal range (2.5-4.5 μL) are associated with increased rates of obesity, elevated TG (Triglycerides) levels, and an increased likelihood of metabolic syndrome (15). However, the popularity of thyroid screening during early pregnancy remains controversial (16).

A systematic review found that serum triglyceride (TG) levels were significantly elevated in all pregnant patients with GDM (17). Other studies also observed that serum TG and cholesterol levels were significantly increased in patients with GDM. The presence of these findings was associated with an increased risk of GDM in early pregnancy (18, 19). High levels of triglycerides (TG) in peripheral blood have been recognized as a characteristic of GDM (20, 21), although this phenomenon is still controversial (22, 23). Importantly, TG levels measured between 9 and 12 weeks of pregnancy had moderate predictive value for subsequent glucose tolerance (24). It can be seen that the lipid level is also of great significance for the early prediction of GDM in high-risk populations.

From the above explanation, both low thyroid hormone levels and high triglyceride levels can increase the risk of GDM, and thyroid hormone levels can affect lipid profiles. However, the network of interactions between them has not been comprehensively reported. Understanding the connection between thyroid and lipids can be helpful in preventing GDM. And most studies have focused on a single controllable factor (obesity) in early pregnancy, with limited effectiveness (25). Therefore, the purpose of this study was to simultaneously explore the effect of thyroid hormone levels and TG levels in early pregnancy on the incidence of GDM.

## Methods

### Patient inclusion criteria

**Figure 1:**
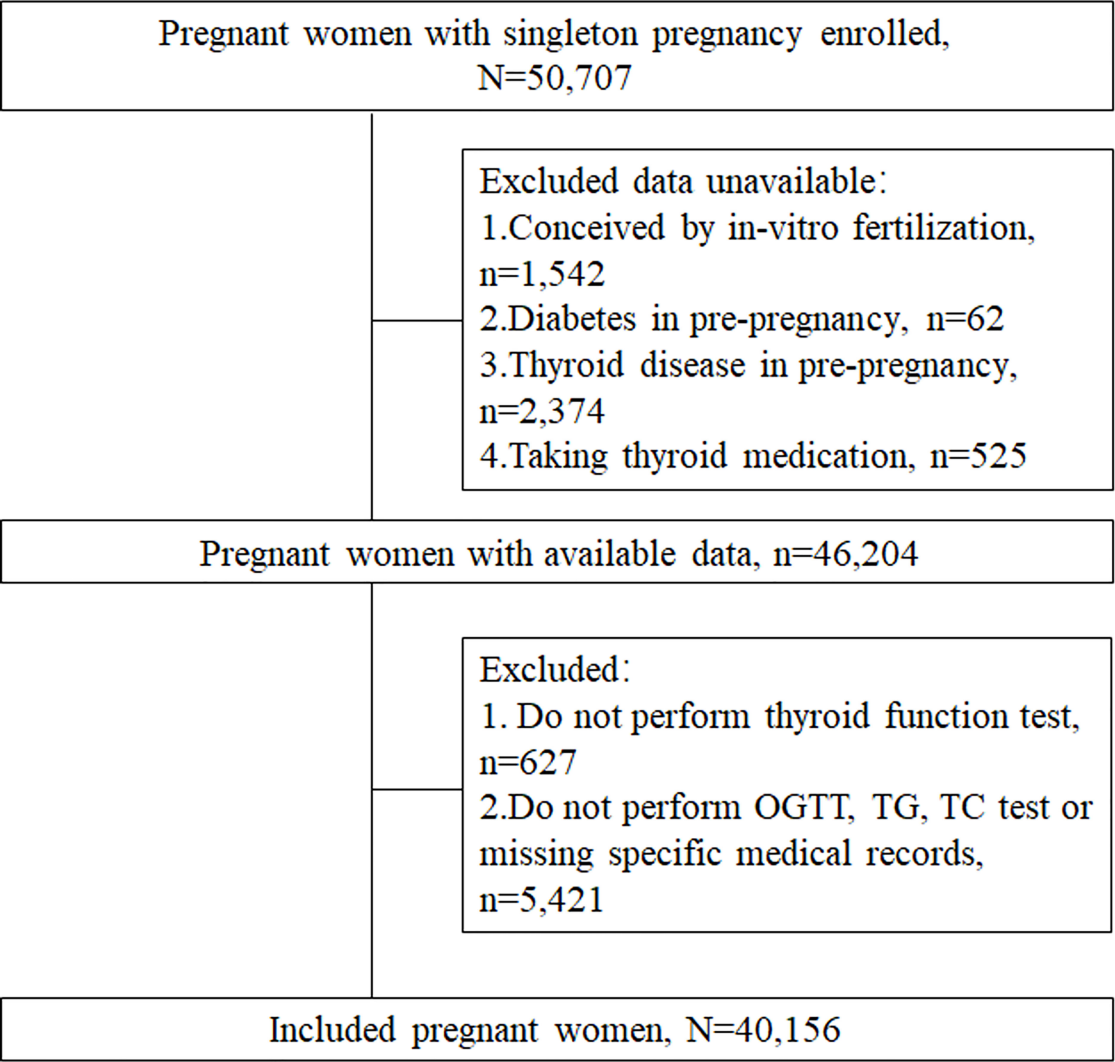
Flowchart of the participants included in the analysis.

Participants in the study were selected from patients enrolled consecutively at the International Peace Maternity and Child Health Hospital (IPMCH). Eligible participants were pregnant women who attended the hospital between January 2013 and December 2017, underwent prenatal tests and had their thyroid function and lipid levels measured during the first trimester of pregnancy. The exclusion criteria were *in vitro* fertilization technology, prepregnancy dyslipidemia, preexisting diabetes before pregnancy, thyroid disease in prepregnancy. Besides, the subjects who were diagnosed to be hypo- or hyperthyroid and other thyroid dysfunction needed medical treatment in the first trimester were also be excluded from the study. Further exclusion criteria were the absence of thyroid function examination, Oral glucose tolerance test (OGTT) and lipid level data (triglycerides, total cholesterol) or the absence of specific medical records (**Figure 1**). Ethical approval was obtained from the Hospital Ethics Committee (GKLW2012-49). Written informed consent was obtained from all study participants.

### Data collection

Nurses, doctors and other medical staff prospectively collected patient data during hospital visits. The first outpatient examination was performed during the first trimester of pregnancy (9-13 weeks of gestation). Doctors used face-to-face interviews to collect data on maternal age, delivery type, education level, and other medical history. The prepregnancy body mass index (BMI) of the pregnant women was calculated from the prepregnancy height measured by nurses and prepregnancy weight reported by the patients.

### Biochemical analysis

The kit (ARCHITECT I2000; Abbott, Chicago, IL) was used to quantify TSH, FT4, Triiodothyronine 3 (T3), Triiodothyronine 4 (T4), and TPOAb concentrations according to the kit protocol. The lower limits of detection and the intra- and inter-assay coefficients of variation were 0.0038 mIU/L and 1.6% and 3.59%, respectively, for TSH; 0.6200 pmol/L and 1.9% and 4.01%, respectively, for FT4; and 1.0 IU/mL and 10% for TPOAb. Euthyroid and thyroid disease entities in early pregnancy were defined by trimester-specific and population based cutoff values using the 2.5th and 97.5th percentile (11.7 and 19.6 pmol/L for FT4, 0.03 and 3.63 mU/L for TSH in early pregnancy) in pregnant women. A TPOAb concentration ≥ 5.61 IU/mL was considered positive. The normal range of FBG and HbA1c is 3.1-5.1 mmol/L, 4.0-6.0%, respectively.

Fasting blood glucose (FBG), serum glycated hemoglobin (HbA1c) and lipid profiles [serum total cholesterol (TC) and triacylglycerol (TG)] were measured in 9-13 weeks of gestation. TC and TG concentrations were determined by solid phase, double locus and chemiluminescence immunoassays using the Immulite 2000 XPi System (Siemens Healthcare Diagnostics, Deerfield, Ill.). The intrabatch coefficients of variation were 8.0%, 6.3% and 5.1% at 9.7, 53.1 and 821.5 IU/L, respectively. FBG concentrations were measured using a GOD PAP kit (Wiesbaden, Germany). The coefficient of variation was 4.3%, and the interbatch coefficient was 3.4%. HbA1c levels were measured by a Roche Diagnostic HbA1c kit (Cobas Integra 800; Roche Diagnostics, Mannheim, Germany) with intrabatch and interbatch coefficients of variation of 2.3% and 2.2%, respectively.

### Diagnostic criteria

GDM screening was conducted in 24–28 weeks of gestation using oral glucose tolerance tests (OGTT). During the test, pregnant women were given 75 g of glucose after an overnight fasting, and blood glucose levels were measured before and at 1 and 2 h after oral administration. The GDM diagnostic criteria using recommendations from the International Association of Diabetes in Pregnancy were a fasting glucose concentration (0h-OGTT) ≥92 mg/dL (5.1 mmol/L), 1-hour glucose concentration (1h-OGTT) ≥180 mg/dL (10.0 mmol/L), and/or 2-hour glucose concentration (2h-OGTT) ≥153 mg/dL (8.5 mmol/L).

### Statistical analysis

Descriptive statistics were tabulated for all variables. Continuous variables are presented as the mean and standard deviation (SD) or median and quartiles. Categorical data are presented as frequencies and percentages. We used ordinary least squares regression models to estimate the relationship between TC, TG and thyroid hormone levels and blood glucose or HbA1c levels. Odds ratios (ORs) and their associated 95% confidence intervals (CIs) derived from multivariable logistic regression models were applied to investigate the associations of TG, TC and thyroid hormone levels with GDM. To assess potential nonlinearity, we applied restricted cubic splines utilizing three knots (10th, 10th-90th and 90th percentiles). All models were adjusted for maternal age, education level, prepregnancy BMI, parity, family history of diabetes and TPOAb status. Independent variables were added to the model as continuous variables.

We evaluated the interaction effects of maternal TG, TC, TSH and FT4 concentrations on blood glucose levels or GDM risk using a generalized linear regression model. A heatmap was constructed to display GDM risk according to combinations of FT4, TSH, TG and TC concentrations, where red indicated a higher probability of GDM and blue indicated a lower probability of GDM. In the interaction effect analysis, a P value less than 0.05 was considered statistically significant.

Mediation analysis was performed to investigate the potential mediation effects of TG levels on the association of FT4 levels with GDM. We used the “mediation” package to calculate the mediating effect with adjustment for maternal age, education level, family history of diabetes, parity and prepregnancy BMI. Other potential confounders such as marital, smoking, alcohol drinking and TPOAb status were not statistically significant in the multivariable regression model which not included in the mediation analysis. Based on the prerequisites that the relationships between the exposure and mediator variables and the outcomes are all statistically significant, the total effect was separated into average direct effects (ADEs) and average causal mediation effects (ACMEs). The total effect was the effect of FT4 levels on GDM, and the ACME was the effect mediated through maternal TG levels. The mediation proportion was obtained by calculating the ACME divided by the total effect.

All statistical analyses were performed using *R v3.6.0* (R Foundation for Statistical Computing, Vienna), using the packages *visreg, rms*, and *mediation* or SPSS Statistics for Windows v20.0 (IBM Corp., Armonk, NY), with a P value < 0.05 considered to be statistically significant.

## Results

After exclusion, this study included 40,156 pregnant women (**Figure 1**). In all, the median (interquartile range [IQR]) maternal age was 30 (28–31) years, the median BMI was 20.6 (IQR: 19.2-22.4) kg/m^2^, and 4819 (12.0%) mothers were diagnosed with GDM. Pregnant women with GDM had higher rates of multiparous, higher education levels and higher rates of diabetes family history.

GDM mothers had lower FT4 and higher TG levels compared with non-GDM mothers in early pregnancy: the median of FT4 levels in GDM and non-GDM mothers were 14.5 (IQR, 13.4-15.8) pmol/L and 14.8 (IQR, 13.7-16.0) pmol/L, respectively; the median of TG levels in GDM and non-GDM mothers were 1.4 (IQR, 1.1-1.9) mmol/L and 1.2 (IQR, 0.9-1.6) mmol/L, respectively. The isolated hypothyroxinemia rate was higher in pregnant women with GDM than in those without GDM (4.0% vs. 2.1%; *P <*0.001). However, the incidence of GDM was not related to TPOAb positive status (10.6% vs. 10.0; *P*=0.22) (**Table 1**).

**Table 1:** Demographic characteristics of the study population.

Maternal characteristics	Total	GDM	Non-GDM	*P*
Age, median (IQR), years	30 (28-31)	31 (28-34)	30 (27-32)	<0.001
BMI, median (IQR), kg/m^2^	20.6 (19.2-22.4)	21.6 (19.9-23.7)	20.5 (19.1-22.3)	<0.001
Multiparous, *n* (%)	7731 (19.3)	1067 (22.1)	6664 (18.9)	<0.001
Education levels, *n* (%)				<0.001
High school and lower	9040 (22.5)	1223 (25.4)	7816 (22.1)	
College and higher	31116 (77.5)	3595 (74.6)	27521 (77.9)	
Marital status, *n* (%)				0.02
Married	35337 (88.0)	4810 (99.8)	35190 (99.6)	
Unmarried	4819 (12.0)	9 (0.2)	147 (0.4)	
Smoking, *n* (%)	33 (0.1)	5 (0.1)	28 (0.1)	0.58
Alcohol drinking, *n* (%)	4819 (12.0)	0 (0.0)	4 (0.0)	0.60
Family history of diabetes, *n* (%)	2676 (6.7)	531 (11.0)	2145 (6.1)	<0.001
FBG in 1st trimester, median (IQR), mmo/L	4.4 (4.2-4.7)	4.6 (4.3-4.9)	4.4 (4.2-4.7)	<0.001
HbA1c in 1st trimester, median (IQR), mmol/mol	32.2 (30.0-33.3)	33.3 (31.1-35.5)	31.1 (30.0-33.3)	<0.001
OGTT test in 2nd trimester				
0h-OGTT, median (IQR), mmol/l	4.1 (3.9-4.4)	4.5 (4.1-4.9)	4.1 (3.8-4.3)	<0.001
1h-OGTT, median (IQR), mmol/l	7.7 (7.1-8.6)	10.2 (9.3-10.9)	7.6 (7.0-8.2)	<0.001
2h-OGTT, median (IQR), mmol/l	6.2 (5.4-7.2)	8.6 (7.5-9.3)	6.1 (5.3-6.9)	<0.001
Maternal lipids in 1st trimester				
TG, median (IQR), mmol/L	1.2 (1.0-1.6)	1.4 (1.1-1.9)	1.2 (0.9-1.6)	<0.001
TC, median (IQR), mmol/L	4.7 (4.2-5.2)	4.7 (4.2-5.2)	4.7 (4.2-5.2)	0.81
Maternal thyroid hormones in 1st trimester				
TSH, median (IQR), mU/L	1.2 (0.7-1.8)	1.1 (0.7-1.7)	1.2 (0.7-1.8)	0.001
FT4, median (IQR), pmol/L	14.7 (13.6-16.0)	14.5 (13.4-15.8)	14.8 (13.7-16.0)	<0.001
T3, median (IQR), nmol/L	2.2 (2.0-2.5)	2.3 (2.1-2.6)	2.2 (2.0-2.4)	<0.001
T4, median (IQR), nmol/L	121.5 (106.6-137.1)	119.6 (104.2-136.5)	121.7 (106.8-137.3)	0.04
TPOAb positive, *n* (%)	4049 (10.1)	510 (10.6)	3539 (10.0)	0.22
Isolated hypothyroxinemia, *n* (%)	939 (2.3)	194 (4.0)	745 (2.1)	<0.001

BMI, body–mass index; FBG, Fast blood glucose; GDM, gestational diabetes mellitus; TG, triglyceride; TC, total cholesterol; HbA1c, hemoglobin A1c; 0h-OGTT, fasting level in the oral glucose tolerance test at 24–28 weeks; 1h-OGTT, 1-hour level in the oral glucose tolerance test at 24–28 weeks; 2h-OGTT2, 2-hour level in the oral glucose tolerance test at 24–28 weeks, IQR, interquartile range.

### Association of maternal TG and FT4 concentrations with GDM in the first trimester

**Figure 2:**
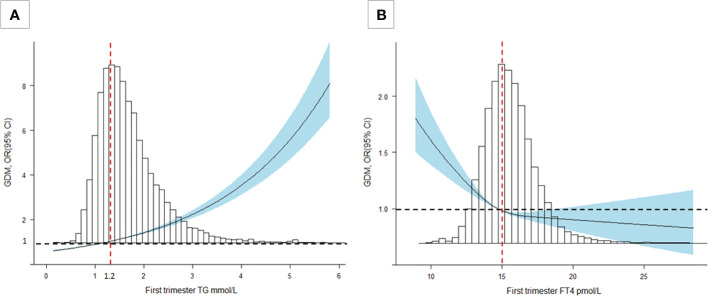
Cubic spline regression analysis of TG and FT4 levels with GDM risk. **(A)** A TG level of 1.2 mmol/L was selected as the reference level (*P*<0.001). **(B)** An FT4 level of 15 pmol/L was selected as the reference level (*P*<0.001). The lines indicate the estimated ORs, and the light blue-shaded areas represent the 95% CIs. All analyses were adjusted for maternal age, education level, family history of GDM, parity, TPOAb status, and prepregnancy BMI.

After adjusting for all covariates, our data suggested TG during the first trimester was positively associated with FBG and HbA1c (*P*<0.001). Similar associations were found in the second trimester for 0h-OGTT (*β*, 0.06; 95% CI: 0.05-0.07), 1h-OGTT (*β*, 0.25; 95% CI: 0.23-0.28) and 2h-OGTT (*β*, 0.37; 95% CI: 0.35-0.39) (**Table 2**). As shown in **Figure 2A**, an increased risk for GDM was associated with increasing TG levels (*P*<0.001): the multivariate OR was 1.58 (95% CI, 1.51-1.65). There was no significant association between TC levels and GDM in the second trimester (**Table 2**).

**Table 2:** Association of triglycerides and serum total cholesterol levels with glucose measurements and gestational diabetes mellitus risk.

	TG		TC	
	β (95% CI)	*P*	β (95% CI)	*P*
First trimester tests				
FBG	0.02 (0.01 to 0.04)	<0.001	0.01 (0.003 to 0.02)	0.01
HbA1c	0.03 (0.01 to 0.04)	<0.001	0.02 (0.01 to 0.03)	<0.001
Second trimester tests				
0h-OGTT	0.06 (0.05 to 0.07)	<0.001	-0.006 (-0.01 to 0.002)	0.16
1h-OGTT	0.25 (0.23 to 0.28)	<0.001	-0.04 (-0.05 to -0.02)	<0.001
2h-OGTT	0.37 (0.35 to 0.39)	<0.001	0.01 (-0.006 to 0.03)	0.20
GDM, OR 95% CI	1.58 (1.51 to 1.65)	<0.001	1.02 (0.98 to 1.06)	0.46

β, The beta coefficients derived from multivariable linear models. Multivariable linear models were adjusted for maternal age, education level, family history of diabetes, parity, and prepregnancy BMI.

FT4 during the first trimester was negatively associated with FBG and HbA1c (*P*<0.001). We also found the negative association in the second trimester for 0h-OGTT (*β*, -0.009; 95% CI: -0.012 to -0.006), 1h-OGTT (*β*, -0.077; 95% CI: -0.084 to -0.070) and 2h-OGTT (*β*, -0.028; 95% CI: -0.034 to -0.021) (**Table 3**). As shown in **Figure 2B**, there was a steep rise in GDM risk with declining maternal FT4 levels (*P*<0.001), especially levels below 15.0 pmol/L. However, higher maternal FT4 concentrations beyond 15.0 pmo/L did not confer additional benefits. The OR of GDM was 0.44 (95% CI, 0.35-0.66) when the FT4 level increased by 1.5 units. No associations with GDM risk were found for TPOAb(+/-) during pregnancy (**Table 3**).

**Table 3:** Association of thyroid hormone levels with glucose measurements and gestational diabetes mellitus risk.

	FT4		TSH		TPOAb (+/-)	
	β (95% CI)	*P*	β (95% CI)	*P*	β (95% CI)	*P*
First trimester tests						
FBG	-0.040 (-0.036 to -0.043)	<0.001	-0.093 (-0.111 to -0.074)	<0.001	0.003 (-0.017 to 0.029)	0.496
HbA1c	-0.036 (-0.031 to -0.040)	<0.001	-0.071 (-0.093 to -0.048)	<0.001	0.017 (-0.011 to 0.046)	0.232
Second trimester tests						
0h-OGTT	-0.009 (-0.012 to -0.006)	<0.001	-0.009 (-0.025 to 0.007)	0.275	0.008 (-0.013 to 0.028)	0.468
1h-OGTT	-0.077 (-0.084 to -0.070)	<0.001	0.012 (-0.025 to 0.049)	0.509	-0.007 (-0.054 to 0.039)	0.757
2h-OGTT	-0.028 (-0.034 to -0.021)	<0.001	-0.092 (-0.127 to -0.057)	<0.001	-0.018 (-0.061 to 0.026)	0.434
GDM, OR 95% CI	0.44 (0.35 to 0.56)	<0.001	0.91 (0.83 to 0.99)	0.036	1.05 (0.95 to 1.16)	0.357

β: The beta coefficients derived from multivariable linear models. Multivariable linear models were adjusted for maternal age, education level, family history of diabetes, parity, TPOAb status, and prepregnancy BMI. a. FT4 and TSH were unnormal distribution, we calculated ln (FT4) and ln (TSH+1) in the model.

**Figure 3:**
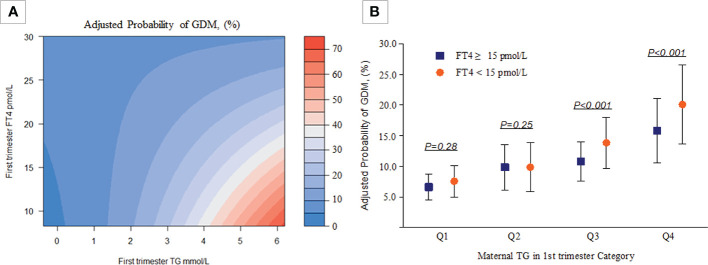
Combined effect of maternal FT4 status and maternal TG categories in early pregnancy on the proportion of women diagnosed with GDM. **(A)** A heatmap for the association of GDM (red colour indicates a higher probability of GDM, blue colour indicates a lower probability of GDM according to the TG-FT4 interaction (*P* for interaction=0.036). **(B)** The y-axis presents adjusted probabilities and 95% CIs of GDM in different TG and FT4 categories (Q1: *P*=0.28, Q2: *P*=0.25, Q3 & Q4: *P*<0.001). All analyses were estimated from a logistic regression model with adjustment for the previously mentioned covariables.

As shown in **Table 4** and **Figure 3**, there was a significant interaction effect of maternal FT4 and TG concentrations on the risk for GDM (*P* for interaction = 0.036). Women in early pregnancy with low FT4 and high TG concentrations had a 2.44-fold increased risk (95% CI, 2.14-2.80) for GDM compared with pregnant women who had relatively adequate FT4 levels (≥15 pmol/L) and low TG levels. Given low maternal FT4 levels, pregnant women in early pregnancy with low or medium TG levels did not have an increased risk for GDM compared with pregnant women with high TG levels (OR, 1.09; 95% CI, 0.93-1.28, *P* = 0.28; OR, 1.09, 95% CI, 1.00-1.20, *P* = 0.28). However, there was no significant interaction between FT4×TC, TSH×TG, or TSH×TC (*P >*0.05) (**Table S1**).

**Table 4:** Combined effect of maternal free triiodothyronine 4 and triglycerides categories on gestational diabetes mellitus risk.

Biochemical characteristics	GDM (%)	Model 1		Model 2	
		Unadjusted OR (95% CI)	*P*	Adjusted OR (95% CI)	*P*
TG, Quartiles					
Q1	697 (7.1)	Ref.		Ref.	
Q2	979 (9.8)	1.43 (1.29-1.58)	<0.001	1.40 (1.26-1.55)	<0.001
Q3	1267 (12.4)	1.86 (1.69-2.05)	<0.001	1.78 (1.61-1.96)	<0.001
Q4	1876 (18.4)	2.95 (2.69-3.23)	<0.001	2.68 (2.44-2.94)	<0.001
*P for trend*	<0.001				
TG binary					
<1.2, mmol/L	1507 (8.2)	Ref.		Ref.	
≥1.2, mmol/L	3312 (15.2)	2.01 (1.89-2.14)	<0.001	1.72 (1.61-1.84)	<0.001
FT4, Quartiles					
Q1	1346 (14.3)	Ref.		Ref.	
Q2	1224 (12.5)	0.86 (0.79-0.93)	<0.001	0.91 (0.84-0.99)	<0.001
Q3	1196 (11.3)	0.76 (0.70-0.83)	<0.001	0.84 (0.77-0.91)	<0.001
Q4	1053 (10.2)	0.68 (0.63-0.74)	<0.001	0.77 (0.71-0.84)	<0.001
*P for trend*	<0.001				
FT4 binary					
<15, pmol/L	2885 (13.2)	1.28 (1.21-1.36)	<0.001	1.18 (1.11-1.26)	<0.001
≥15, pmol/L	1934 (10.6)	Ref.		Ref.	
**Interaction effect**					
Low TG (Q1)					
FT4≥15, pmol/L	311 (6.6)	Ref.		Ref.	
FT4<15, pmol/L	386 (7.6)	1.16 (0.99-1.35)	0.07	1.01 (0.87-1.18)	0.87
Medium TG (Q2-Q3)					
FT4≥15, pmol/L	972 (10.3)	1.62 (1.42-1.85)	<0.001	1.50 (1.31-1.72)	<0.001
FT4<15, pmol/L	1274 (11.9)	1.92 (1.68-2.18)	<0.001	1.52 (1.33-1.73)	<0.001
High TG (Q4)					
FT4≥15, pmol/L	651 (15.8)	2.66 (2.31-3.07	<0.001	2.16 (1.87-2.49)	<0.001
FT4<15, pmol/L	1225 (20.1)	3.56 (3.12-4.06)	<0.001	2.44 (2.14-2.80)	<0.001
**Stratified by TG categories**					
Low TG (Q1)					
FT4≥15, pmol/L	311 (6.6)	Ref.		Ref.	
FT4<15, pmol/L	386 (7.6)	1.16 (0.99-1.35)	0.07	1.09 (0.93-1.28)	0.28
Medium TG (Q2-Q3)					
FT4≥15, pmol/L	972 (10.3)	Ref.		Ref.	
FT4<15, pmol/L	1274 (11.9)	1.18 (1.08-1.29)	<0.001	1.09 (1.00-1.20)	0.05
High TG (Q4)					
FT4≥15, pmol/L	651 (15.8)	Ref.		Ref.	
FT4<15, pmol/L	1225 (20.1)	1.34 (1.20-1.48)	<0.001	1.26 (1.13-1.40)	<0.001

Model 1: Unadjusted model; Model 2: Adjusted to maternal age, education level, family history of diabetes, parity, TPOAb status, and prepregnancy BMI.

### Mediation analysis

**Figure 4:**
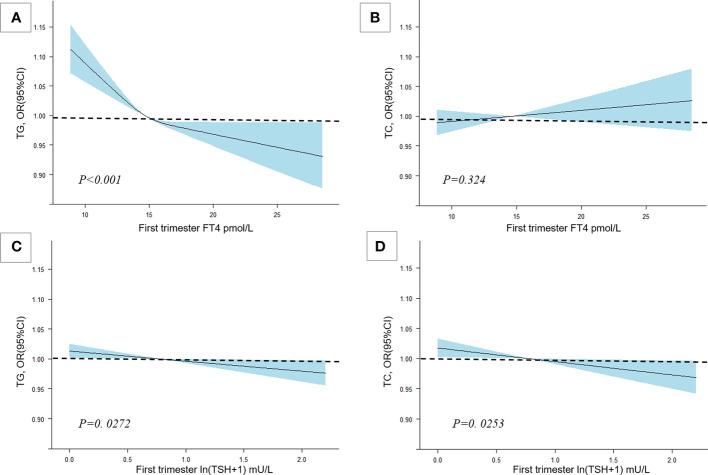
The association among TC, TG and maternal thyroid hormone levels. Cubic spline regression analysis of TG and FT4 levels with thyroid hormone levels. Multivariable RCS models were adjusted for maternal age, education level, family history of diabetes, parity, TPOAb status, and prepregnancy BMI. **(A)** The association between FT4 and TG. **(B)** The association between FT4 and TC. **(C)** The association between ln(TSH+1) and TG. **(D)** The association between ln(TSH+1) and TC.

**Figure 5:**
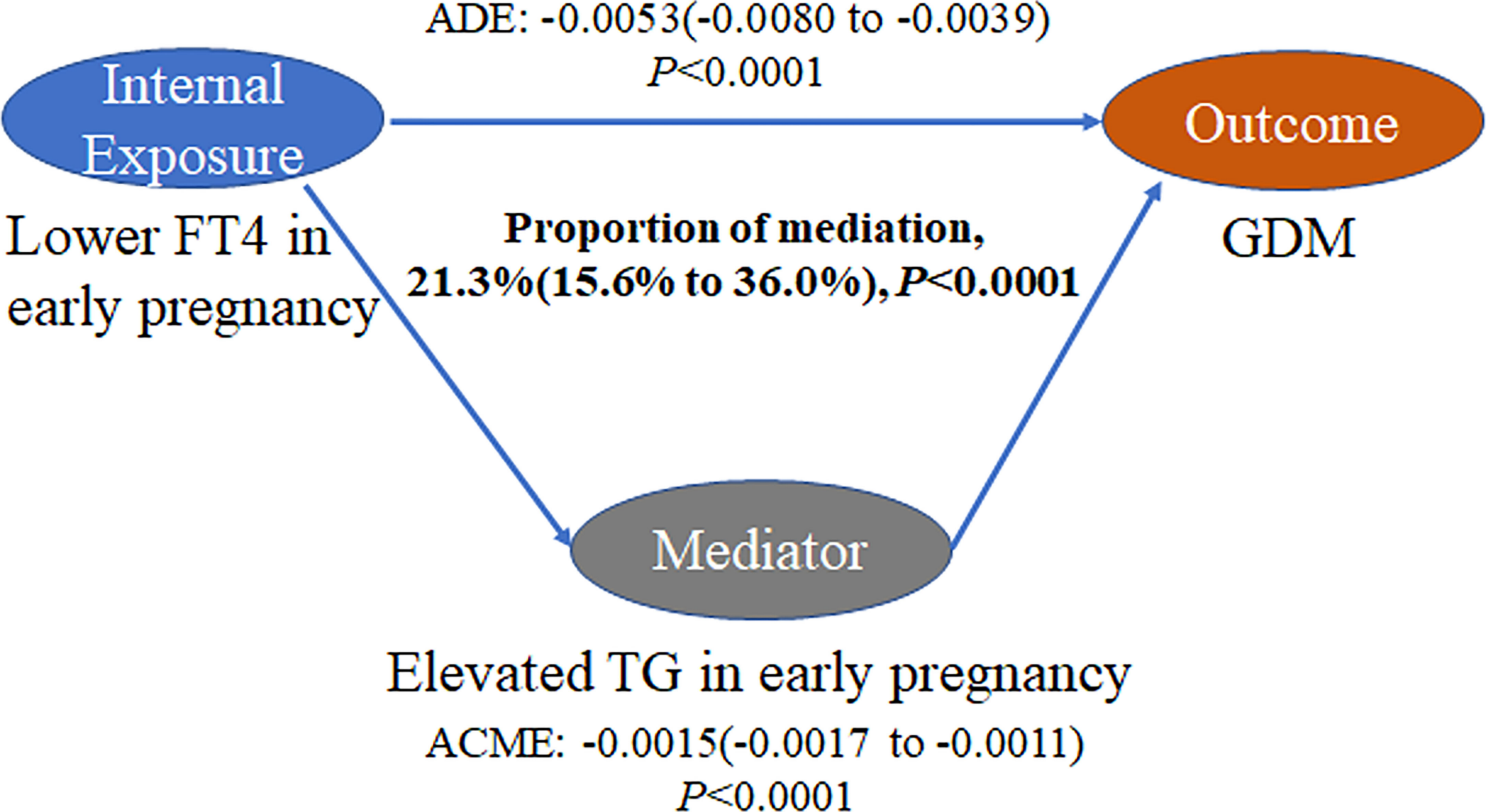
Mediation analysis of the association of FT4 levels with GDM by TG levels. The figure presented TG as the mediator, the estimate of average mediation effects (ACME), the estimate of the average direct effects (ADE), and the proportion of mediation. The mediation models were adjusted for maternal age, education levels, family history of diabetes, parity and prepregnancy BMI.

There was a negative association of FT4 levels with GDM as well as a negative association of TG levels with FT4 levels in the current study population (**Figure 4**). The mediation analysis revealed a total effect of FT4 levels on GDM of -0.0068 (95% CI, -0.0096 to -0.0045; *P* < 0.001), including a significant direct effect of -0.0053 (95% CI, -0.0080 to -0.0039; *P* < 0.001). A statistically significant causal mediating effect of FT4 levels was associated with GDM, in which an average indirect effect of -0.0015 (95% CI, -0.0017 to -0.0011; *P* < 0.001) through maternal TG levels was found, and the estimated proportion of the mediating effect was 21.3% (95% CI, 15.6% to 36.0%; *P* < 0.001) (**Figure 5**). However, we did not find an association of TG levels with GDM mediated by FT4 levels (*P*=0.28) (**Table S2**).

### Sensitivity analyses to assess the robustness of the findings

The mediating effects did not differ appreciably across the following strata: TPOAb status, BMI (normal weight or overweight/obese), parity (primiparous or multiparous), age group (<35 y or ≥35 y), education level (high school and lower or college and higher), or FBG group (<5.1 mmol/L or ≥5.1 mmol/L) (**Table S3**).

## Discussion

Our current study found that higher TG levels and lower FT4 levels in early pregnancy were associated with higher glucose concentrations and a higher risk of GDM shown on OGTT at weeks 24-28. Interactions were observed for TG and FT4 with glucose and GDM, a combination of low FT4 and high TG quartiles had stronger associations with GDM. In addition, we found that 21.3% of the estimated association between FT4 levels and GDM is mediated by TG levels. Having a larger sample size with sensitivity analysis enabled us to explore the complex mediation of TG and FT4 with GDM, and to clarify previously inconsistent findings with greater confidence.

Gestational diabetes mellitus is a common chronic pregnancy disease affecting the health of millions of women worldwide (26, 27). GDM not only causes adverse pregnancy outcomes such as preeclampsia and macrosomia but also increases the risk of developing type 2 diabetes later in life (28, 29). Therefore, we should diagnose and intervene in GDM as early as possible. Kapadia et al. reported that patients with subclinical hypothyroidism had significantly higher insulin resistance indices than controls (30). However, another study showed that higher FT3 levels and FT3/FT4 ratios were associated with increased GDM risk (31). Our study found that lower FT4 levels could increase the risk of GDM.

Another study has found that GDM not only leads to changes in glucose metabolism but is also accompanied by changes in lipid metabolism. GDM is associated with maternal adipose tissue dysfunction, such as increased lipid decomposition, increased maternal TG levels, and decreased HDL and cholesterol levels (32). Other studies have also shown that patients with GDM have higher TG levels (33). In addition, a meta-analysis showed that elevated lipid levels, particularly TG levels, were significantly associated with an increased risk of GDM and could potentially be incorporated into risk stratification algorithms to calculate the risk of GDM (34). These studies are consistent with our findings. Two large cross-sectional studies found elevated serum TC and TG levels in patients with mild hypothyroidism (35, 36). Therefore, we envisage that there may be some connection among FT4 levels, TG levels and GDM. Our results corroborate this point as well.

In our study, we found that 21.3% of the relationship between FT4 levels and GDM was mediated by TG levels, but there was no mediating effect of FT4 levels in the relationship between TG levels and GDM. Biologically, no study has found that dyslipidaemia directly leads to thyroid dysfunction. However, abnormal thyroid function can lead to dyslipidaemia. In hypothyroidism, the main reason for dyslipidaemia is that the synthesis rate is higher than the degradation rate, providing the substrate for lipid peroxidation of reactive oxygen species (ROS), which leads to oxidative stress (37). In addition, the fatty acid synthesis and decomposition rate of patients with hypothyroidism was decreased, and the fat decomposition sensitivity of white adipocytes was decreased (38). The effects of thyroid hormones on triglyceride metabolism include increasing hydrolysis and new lipogenesis through the transcription of several key lipogenic genes (39). Therefore, it is understandable that TG levels have a mediating effect, while FT4 levels do not.

Our study demonstrates an L-type association between maternal FT4 levels and GDM, and low TG levels may be an important mediator in this association. The isolated hypothyroxinemia rate was higher in pregnant women with GDM. Both high levels of TG and low levels of FT4 were associated with a higher risk of GDM, respectively, and normal FT4 levels were critical for the homeostasis of blood lipid levels. Therefore, it can be understood that TG plays an intermediate role in the relationship between GDM and FT4. Our findings suggest that pregnant women with hypothyroidism need careful monitoring of triglyceride changes during the first trimester to reduce the risk of GDM. However, this needs to be further confirmed in clinical studies so that clinicians can better manage all aspects of patients in the future.

Our study suggests that TG and FT4 levels in early pregnancy may be independent and modifiable risk factors for GDM. High TG levels and thyroid dysfunction in early pregnancy are also common for women, which highlights the need to maintain a normal weight and pay attention to personal diet during early pregnancy. Future researchers are encouraged to focus on (1) investigating the effects of establishing healthy TG levels and thyroid hormone levels in early pregnancy on blood glucose levels and the incidence of GDM and/or complications and (2) studying the epigenetic mechanism of TG and FT4 levels on GDM.

The innovation of this study lies in the following aspects. First, this is the first time that TG levels as an intermediary factor were shown to mediate the relationship between FT4 levels and GDM based on a large sample. Second, this study was a prospective study with little information bias, and the results obtained are more reliable than those of a retrospective study. Third, we excluded patients using IVF because pregnant women using assisted reproductive technology are also at increased risk of GDM (40). We also excluded patients with pregestational diabetes and thyroid disease and ruled out drug interference. Finally, we constructed a network of interactions between age, BMI before pregnancy, family history of GDM, education level, parity, FT4 levels and TG levels in early pregnancy and GDM, which can more comprehensively detect the interactions among them. We clarified the stability of the mediating effect of TG levels in each subgroup, indicating that it has a high universality within the population.

However, our study also has limitations. First, this was a single-centre prospective study, and its results may not be applicable to women in other regions. Therefore, future studies should be conducted on a broader population basis to increase the reliability and universality of the results. Second, some patients did not undergo corresponding tests in a timely manner, resulting in missing data, leading to some information bias. Third, to overcome this potential source of bias, we controlled for known confounders by using regression adjustment. In addition, sensitivity analyses were used to assess the potential bias. Despite these efforts, it is possible that in this study, as in all observational studies, unmeasured confounding could have biased the estimates of indirect and direct effects (41, 42). For bias on mediation analysis, it has been found that traditional mediation analysis methods are prone to bias due to incorrect statistical analysis and suboptimal research design. Now new statistical methods have been developed, although some methods are not fully implemented, and in some cases the appropriate methods simply do not exist. The traditional mediation analysis method is still frequently used, and the results of early epidemiological studies using this method should not be discarded (43).

## Conclusions

In conclusion, our study demonstrated that lower FT4 levels in early pregnancy were associated with a higher risk of GDM, which may be mediated by maternal TG concentrations. This study identifies possible underlying mechanisms for the occurrence of GDM, which may provide a reasonable prognosis for the early diagnosis of GDM. It is necessary to maintain TG levels in a lower stage while improving thyroid function in early pregnancy to reduce the risk of GDM.

## Data availability statement

The original contributions presented in the study are included in the article/**Supplementary Material**. Further inquiries can be directed to the corresponding authors.

## Ethics statement

Ethical approval was obtained from the Hospital Ethics Committee (GKLW2012-99 49). Written informed consent was obtained from all study participants.

## Author contributions

HH and XL designed the study. GD, LB and YW supervised data collection. KS conducted statistical analyses. CZ drafted the original version of manuscript. All authors contributed to revision and approved the final version for publication. HH and YW are the guarantors for this work and accept full responsibility for the conduct of the study, had access to the data, and controlled the decision to publish. All authors contributed to the article and approved the submitted version.

## Funding

The authors thank the National Natural Science Foundation of China (82001571, 82088102, 81661128010, 82171686), the National Key Research and Development Program (2021YFC2701601, 2021YFC2700701), CAMS Innovation Fund for Medical Sciences (2019-I2M-5-064), Collaborative Innovation Program of Shanghai Municipal Health Commission (2020CXJQ01), the International Science and Technology Collaborative Fund of Shanghai (18410711800), Program of Shanghai Academic Research Leader (20XD1424100), Clinical Research Plan of SHDC (SHDC2020CR1008A, SHDC12019107, SHDC12018X17) and Shanghai Frontiers Science Research Base of Reproduction and Development.

## Acknowledgments

The authors gratefully acknowledge the contributions and efforts of all pregnant women who participated in this study and the doctors and nurses involved in data collection and patient care. We also acknowledge the following team members, collaborators and other staff who have contributed to this study. School of public health, Fudan University: Tiejun Zhang (principal investigator [PI]), International Peace Maternity and Child Health Hospital, School of Medicine, Shanghai Jiao Tong University: Jianxia Fan (PI), Department of Obstetrics and Gynecology, School of Clinical Sciences at Monash Health, Monash University: Ben W Mol (PI), Clinical Research Unit, Shanghai First Maternity and Infant Hospital, Tongji University School of Medicine: Zhen Li (PI).

## Conflict of interest

The authors declare that the research was conducted in the absence of any commercial or financial relationships that could be construed as a potential conflict of interest.

## Publisher’s note

All claims expressed in this article are solely those of the authors and do not necessarily represent those of their affiliated organizations, or those of the publisher, the editors and the reviewers. Any product that may be evaluated in this article, or claim that may be made by its manufacturer, is not guaranteed or endorsed by the publisher.
