# Supplementary material for: Association of maternal triglyceride responses to thyroid function in early pregnancy with gestational diabetes mellitus
Source: Front Endocrinol (Lausanne). 2022 Nov 28;13:1032705. doi: 10.3389/fendo.2022.1032705 (PMC9742591; doi:10.3389/fendo.2022.1032705)
Supplement: Supplementary file 1 [file Table_1.docx]

Supplementary Material

## Supplementary Tables

| Interaction | 0h-OGTT | | 1h-OGTT | | 2h-OGTT | | GDM | |
| --- | --- | --- | --- | --- | --- | --- | --- | --- |
|  | Coef. (SE) | *P* | Coef. (SE) | *P* | Coef. (SE) | *P* | Coef. (SE) | *P* |
| FT4×TG | -0.0059(0.0027) | 0.03 | -0.016(0.006) | 0.01 | -0.013(0.006) | 0.02 | -0.03(0.01) | 0.03 |
| FT4×TC | -0.0021(0.002) | 0.30 | 0.0011(0.0046) | 0.81 | 0.0017(0.0043) | 0.38 | 0.004(0.010) | 0.69 |
| TSH×TG | 0.0015(0.0057) | 0.79 | 0.0184(0.013) | 0.16 | 0.015(0.012) | 0.21 | 0.046(0.025) | 0.07 |
| TSH×TC | 0.029(0.004) | 0.50 | 0.001(0.010) | 0.91 | 0.013(0.009) | 0.15 | 0.042(0.022) | 0.06 |

Multivariable logistic models were adjusted for maternal age, education level, family history of diabetes, parity, TPOAb status, and prepregnancy BMI.

**Supplementary Table S1.** Interaction effects of thyroid hormone and lipid levels with glucose measurements and GDM risk.

| Mediation | Total effect (CI) | ADE (CI) | ACME (CI) | Proportion of mediation (%) |
| --- | --- | --- | --- | --- |
| FT4-TG-GDM | -0.0068(-0.0096 to -0.0045)** | -0.0053(-0.0080 to -0.0039)** | -0.0015(-0.0017 to -0.0011)** | 21.3(15.6 to 36.0)** |
| TG-FT4-GDM | -0.037(-0.0034 to 0.04) | -0.037(-0.034 to 0.04) | -0.00008(-0.00005 to 0.01) | 0.24(-0.14 to 1.0) |

Multivariable logistic models were adjusted for maternal age, education levels, family history of diabetes, parity, and prepregnancy BMI. **p-value <0.001. ACME, average causal mediation effect; ADE, average direct effect.

**Supplementary Table S2.** Mediation analysis of the association of TG and FT4 levels with GDM.

| Subgroups | GDM(%) | Proportion of mediation (%) | *P* |
| --- | --- | --- | --- |
| TPOAb groups |  |  |  |
| TPOAb(+) | 510(12.6) | 18.4(7.7 to 90.0) | 0.04 |
| TPOAb(-) | 4309(11.9) | 21.4(15.8 to 34.0) | <0.001 |
| BMI |  |  |  |
| Normal weight | 3230(11.1) | 23.1(11.2 to 72.0) | 0.02 |
| Overweight or obese | 1103(23.5) | 22.4(11.7 to 54.0) | <0.001 |
| Age |  |  |  |
| <35 y | 3846(10.9) | 18.5(8.83 to 48.0) | <0.001 |
| ≥35 y | 973(19.5) | 22.6(15.5 to 34.0) | <0.001 |
| Parity |  |  |  |
| Primiparous | 3752(11.6) | 23.0(17.0 to 35.0) | <0.001 |
| Multiparous | 1067(13.8) | 16.4(5.27 to 71.0) | 0.02 |
| Family history of GDM |  |  |  |
| Yes | 531(19.8) | 11.4(3.2 to 41.0) | <0.001 |
| No | 4288(11.4) | 22.9(16.4 to 34.0) | <0.001 |
| Education levels |  |  |  |
| High school and lower | 1223(13.5) | 18.4(11.1 to 29.0) | <0.001 |
| College and higher | 3595(11.6) | 24.6(14.7 to 37.0) | <0.001 |
| FBG |  |  |  |
| <5.1 mmol/L | 3740(10.9) | 23.1(15.7 to 35.0) | <0.001 |
| ≥5.1 mmol/L | 547(31.2) | 11.1(5.2 to 19.0) | <0.001 |

**Supplementary Table S3.** Sensitivity analysis of the association of TG and FT4 levels with GDM.
